# Scanner-generated native T1 mapping: a novel approach for assessing myocardial fibrosis in coronary heart disease

**DOI:** 10.3389/fcvm.2025.1553919

**Published:** 2025-05-01

**Authors:** Li Kong, Xiaohong Tian, Bing Ji, Jian Wang, Hongqin Liang, Xiaojuan Ji

**Affiliations:** ^1^ Department of Ultrasound, Children's Hospital of Chongqing Medical University, National Clinical Research Center for Child Health and Disorders, Ministry of Education Key Laboratory of Child Development and Disorders, Chongqing Engineering Research Center of Stem Cell Therapy, Chongqing, China; ^2^ Department of Radiology, Southwest Hospital, Army Medical University (Third Military Medical University), Chongqing, China; ^3^ Department of Ultrasound, Chongqing General Hospital, Chongqing, China

**Keywords:** hematocrit, invasive coronary angiography, ECV, coronary heart disease, CMR, PACS

## Abstract

**Background:**

To investigate the feasibility of using native longitudinal relaxation time (T1) mapping values, derived from the Picture Archiving and Communication System (PACS), for assessing diffuse myocardial fibrosis in patients with coronary heart disease (CHD).

**Materials and methods:**

Patients with CHD group were retrospectively enrolled as the experimental group, while age- and sex-matched healthy individuals were included as the control group. Based on the results of late gadolinium enhancement (LGE) sequence from cardiac magnetic resonance (CMR) imaging, the CHD group was further stratified into two subgroups: the LGE positive group (LGE+) and the LGE negative group (LGE−). The correlation between native T1 values and extracellular volume (ECV) values were assessed using the Pearson correlation coefficient.

**Results:**

A total of 60 patients with coronary heart disease (age 54.03 ± 9.86 years) were included in the analysis, of whom 30 had late gadolinium enhancement (LGE+) and 30 did not (LGE−). The control group consisted of 42 healthy subjects (age 52.14 ± 7.41 years). Compared with the control group, both native T1 and extracellular volume (ECV) values were significantly increased in the CHD group (*P* < 0.05). The native T1 value was positively correlated with the ECV value (*r* = 0.711, *P* < 0.01). In the LGE+ subgroup, native T1 and ECV values were significantly higher than those in the control group (*P* < 0.001). The area under the receiver operating characteristic curve (AUC) for native T1 was 0.763. The optimal diagnostic threshold for native T1, as measured by the Picture Archiving and Communication System (PACS), was 1,275.50 ms, with a sensitivity of 93.3% and a specificity of 63.3%.

**Conclusions:**

The diagnostic performance of scanner-generated native T1 Mapping demonstrates robust accuracy and holds potential as a non-invasive tool for evaluating diffuse myocardial fibrosis in patients with CHD.

## 1 Introduction

In recent years, the incidence of coronary heart disease (CHD) has remained high, and the affected population is becoming increasingly younger (1, 2). CHD is mainly associated with two different myocardial pathologies, including focal myocardial injury, known as myocardial infarction and diffuse injury (3–5). Both types of injury are closely related to adverse cardiovascular events (6, 7). Diffuse fibrosis was often overlooked due to its subtle clinical symptoms. The gold standard for detecting myocardial fibrosis was histopathological analysis of tissue samples. However, due to its invasive nature and sampling errors, it was not widely applicable in routine clinical practice (8).

Cardiac magnetic resonance imaging (CMR) played a crucial role in evaluating different degrees of myocardial edema and fibrosis as a non-invasive imaging modality (9, 10). Recently, late gadolinium enhancement (LGE) on CMR has become the gold standard for diagnosing focal myocardial infarction. However, it is less effective in differentiating diffuse myocardial fibrosis (11–13). Previous studies have shown that extracellular volume (ECV) based on CMR longitudinal relaxation time (T1) mapping technology can serve as a quantitative marker for diffuse fibrosis, showing strong correlation with histology (14–16). However, the calculation of ECV requires the acquisition of hematocrit (HCT) and the use of specialized cardiac post-processing software, which limits its clinical application (17, 18). Thus, it is urgent to find a simpler method.

The Picture Archiving and Communication System (PACS) image workstations are computer systems used primarily by diagnosticians to view, diagnose, and analyze images. The images generated by the scanner can be transmitted to the PACS via the network. Previous studies have reported that T1 value measured on PACS workstations can be used to assess diffuse myocardial fibrosis in patients with non-ischemic cardiomyopathies, such as hypertrophic cardiomyopathy (19, 20). However, its application in the assessment of diffuse myocardial fibrosis in patients with common coronary heart disease is rare. If this approach is feasible, radiologists would not need to purchase additional expensive cardiac post-processing software to assess chronic diffuse myocardial fibrosis in patients with CHD, thereby saving costs and simplifying the workflow.

Based on this, the main purpose of this study is to compare the consistency and correlation between the average native T1 value measured by PACS and the ECV values calculated by dedicated cardiac software. This comparison aims to explore the feasibility and advantages of using **scanner-generated** native T1 values for the assessment of diffuse myocardial fibrosis in patients with CHD on PACS workstations.

## 2 Methods

### 2.1 Study population

The retrospective study was conducted in accordance with the Declaration of Helsinki (as revised in 2013). This study was approved by and registered with the Medical Science Research Ethics Committee, and individual consent for this retrospective analysis was waived. Inclusion criteria enquired participants to (a) be diagnosed with stable CHD after undergoing an invasive coronary angiography (ICA), (b) conducted CMR examination within two weeks before or after ICA. Exclusion criteria included (a) other heart diseases (hypertensive cardiomyopathy, diabetes, etc.), (b) presence of congenital heart disease or valvular disease, and (c) concomitant conduction block or arrhythmia.

### 2.2 Cardiac magnetic resonance image

All subjects underwent 3.0 T magnetic resonance imaging (MRI, MAGNETOM Trio, Siemens Healthcare, Erlangen, Germany). Applied balanced steady-state free precession (bSSFP) sequence to acquire cine images including the short axis, long axis, four-chamber, and two-chamber views. The scan parameters were as follows: slice thickness = 6 mm, echo time = 1.7 ms, field of view = 325 mm × 400 mm, matrix = 256 × 256, and in-plane voxel size = 1.5 mm. A breath-hold, ECG-gated modified Look-Locker inversion recovery (MOLLI) sequence with patterns of 5b(3b)3b was utilized to obtain native and post T1 mapping at the three short-axis levels of the left ventricle (basal, middle, and apex). The scanning parameters were as follows: FOV = 360 mm × 300 mm, matrix = 169 × 256, TR = 324.9 ms, TE = 1.1 ms; minimum inversion time = 120 ms, inversion angle = 35°, and acceleration factor =  2. The injection dose of gadoteric acid meglumine was 2.0 ml/kg with a flow rate of 3.0 ml/s via the elbow vein. After injection, 10 ml of normal saline was injected as a flush. LGE imaging of the myocardium was performed 10 min later using a phase-sensitive inversion recovery (PSIR) prepared fast low-angle shot (FLASH) sequence. The scanning parameters were as follows: TE = 1.93 ms, TR = 500–800 ms, slice thickness = 8 mm, FOV = 300 mm × 400 mm, matrix = 256 × 140.

### 2.3 Image analysis

We imported the images into the CVI42 (Circle Cardiovascular Imaging Inc., Calgary, Alberta, Canada) post-processing software to observe cardiac function parameters. The endocardial and epicardial boundaries of the left ventricle were meticulously delineated at each level on the short-axis 3D module, excluding papillary muscles, intracavitary blood pools, and epicardial adipose tissue, to accurately obtain traditional cardiac functional parameters. These parameters encompassed left ventricular ejection fraction (LVEF), left ventricular end systolic volume (LVESV), left ventricular end diastolic volume (LVEDV), left ventricular stroke volume (LVSV), cardiac index (CI), and cardiac output (CO). In the T1 mapping module, the endocardial and epicardial boundaries of the left ventricle were separately delineated on both native T1 and post-contrast T1 maps, employing the same methodology as described for the short-axis 3D module.

Afterwards, we calculated synthetic HCT values using the formula Synthetic HCT = [562 * (1/T1blood)] + 0.098 (21). Finally, we determined the overall left ventricular ECV using the formula, ECV = (1 − HCT) * (1/T1enhancement myocardium − 1/T1native myocardium)/(1/T1 enhancement blood pool − 1/T1native blood pool) (22).

The measurements of scanner-generated native T1 were performed on the PACS workstation (INFINITT Healthcare, Seoul, South Korea) using the 17-segment analysis method recommended by the American Heart Association (AHA) (23). The native T1 values of the myocardial basal, mid, and apical layers were measured across a total of 16 segments. For each segment, three regions of interest (ROIs) were selected, with each ROI having an area ranging from 2.50 to 3.50 mm^2^ (24). The values for each segment or layer were derived by averaging the mean values of the ROIs defined within their respective ranges.

### 2.4 Statistical analysis

All statistical analyses were performed using the commercial software SPSS (Version 25; IBM Corp., Armonk, NY, USA). The normality of measurement data was assessed using the Shapiro–Wilk test. Normally distributed measurement data was presented as mean ± standard deviation $\left(\bar{X}\pm S\right)$, and the independent sample *t*-test was applied for group comparisons. For non-normally distributed measurement data, median (interquartile range) was used, and the non-parametric. The Pearson correlation coefficient was used to examine the correlation between native T1 and ECV image parameters in each group. Receiver operating characteristic (ROC) curves were generated for native T1 value to determine the optimal thresholds, specificity, and sensitivity. The intra-class correlation coefficient (ICC) was used to assess the consistency of delineated native T1 values. *P* < 0.05 was considered statistically significant.

## 3 Results

### 3.1 Participants characteristics

**Figure 1:**
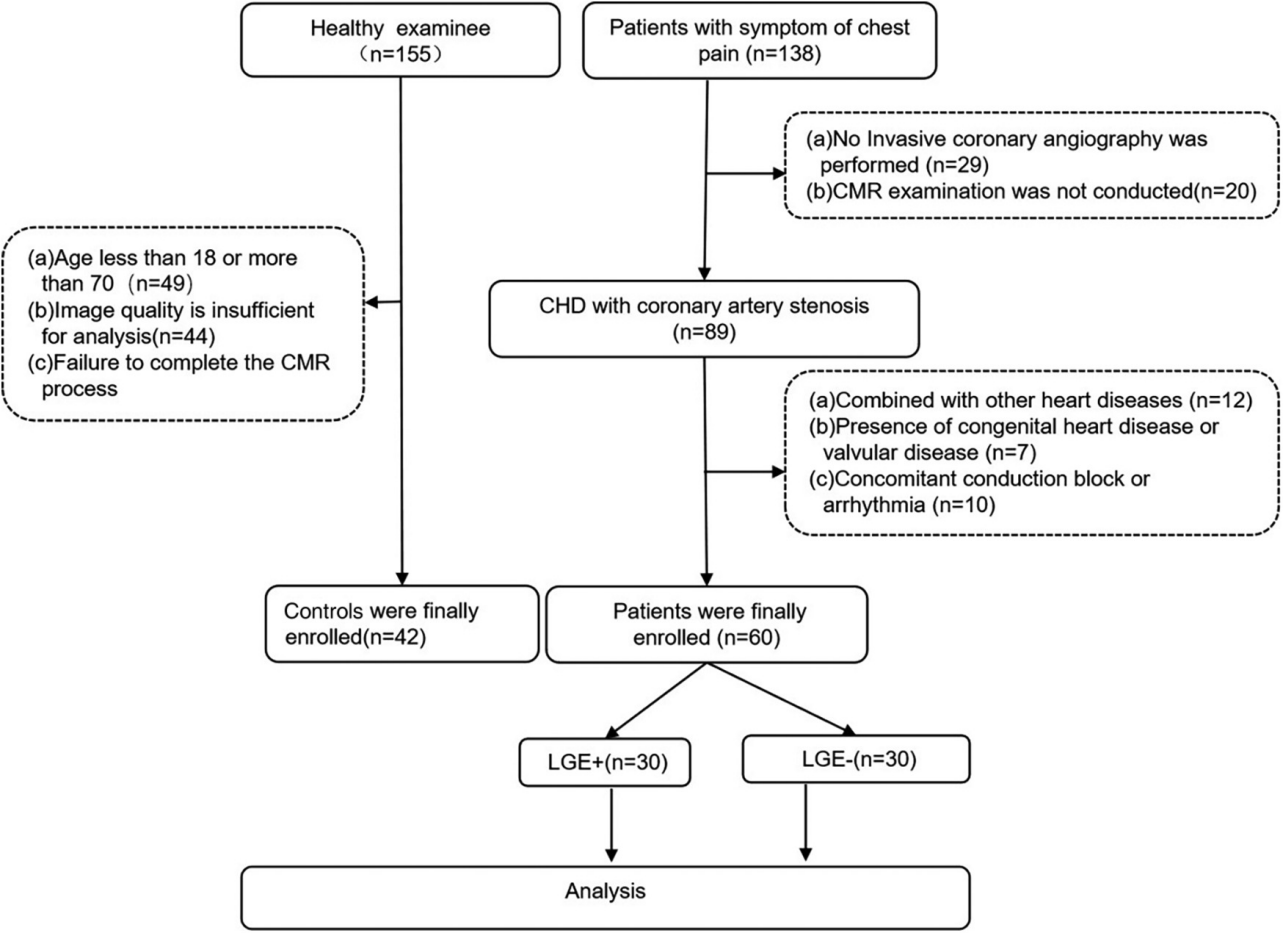
The flowchart of study population.

In the CHD group, a total of 60 patients (54.03 ± 9.86 years old) were included in the analysis, and 42 patients in the control group (52.14 ± 7.41 years old). The flow chart is shown in Figure 1. There were no significant differences in age, sex, height, weight, systolic blood pressure, and diastolic blood pressure between the CHD group and the control group, as well as between the LGE+ and LGE-groups. (*P* > 0.05) (Tables 1, 2).

**Table 1:** The demographic characteristics of CHD group and control group.

Parameters	CHD (*n* = 60)	Control (*n* = 42)	*P*	*t*
Age (year)	54.03 ± 9.86	52.14 ± 7.41	0.06	3.28
Gender (male/total)	43 (71%)	21 (50%)	0.11	−2.50
Height (mm)	164.54 ± 6.55	162.38 ± 8.09	0.14	1.49
Weight (kg)	67.95 ± 11.17	65.26 ± 8.74	0.20	1.30
Systolic pressure (mmHg)	77.30 ± 10.06	75.52 ± 7.37	0.33	1.00
Diastolic pressure (mmHg)	121.5 ± 14.67	116.67 ± 10.35	0.70	1.84

**Table 2:** Patient demographic characteristics of LGE+ group and LGE− group.

Parameters	LGE+ (*n* = 30)	LGE− (*n* = 30)	*P*	*t*
Age (year)	53.53 ± 8.7	55.53 ± 10.8	0.24	−1.18
Gender (male/total)	15 (50%)	15 (50%)	0.40	−0.85
Height (mm)	165.32 ± 6.05	163.77 ± 7.01	0.36	0.92
Weight (kg)	68.38 ± 10.77	67.52 ± 11.73	0.77	0.30
Systolic pressure (mmHg)	77.33 ± 11.62	11.27 ± 8.42	0.98	0.03
Diastolic pressure (mmHg)	120.73 ± 15.17	122.27 ± 14.35	0.69	−0.40

### 3.2 Comparison of cardiac function, native T1 value and ECV between CHD group and control group

There was no statistical significance in LVEDV, CO and CI between CHD group and control group (*P* > 0.05). In the CHD group, the values of LVESV, ECV, and scanner-generated native T1 were all higher than those in the control group, while the LVSV and LVEF were lower (*P* < 0.05). The ECV value of the CHD group and the control group were 29.76 ± 2.7 and 28.00 ± 3, and the scanner-generated native T1 value were 1,286.03 ± 35.15 and 1,256.16 ± 30.9, respectively (Table 3).

**Table 3:** Comparison of cardiac function, native T1 value and ECV between CHD group and control group.

Parameters	CHD (*n* = 60)	Control (*n* = 42)	*P*	*t*
LVEDV (ml, $\bar{x}\pm s$)	114.86 ± 33.04	105 ± 16.33	0.08	1.79
LVESV (ml, $\bar{x}\pm s$)	52.76 ± 26.12	41 ± 9.09	<0.01**	3.20
LVSV (ml, $\bar{x}\pm s$)	61.93 ± 15.84	67.88 ± 13.87	0.05*	−1.97
CO (L/min, $\bar{x}\pm s$)	4.40 ± 1.22	4.70 ± 1.21	0.22	−1.20
CI (L/min/m^2^, $\bar{x}\pm s$)	2.52 ± 0.82	2.72 ± 0.63	0.19	−1.30
LVEF (%, $\bar{x}\pm s$)	54.3 ± 10.14	61.17 ± 5.32	<0.01**	−4.44
ECV (%, $\bar{x}\pm s$)	29.76 ± 2.70	28.00 ± 3.00	<0.01**	3.06
Native T1 (ms, $\bar{x}\pm s$)	1,286.03 ± 35.15	1,256.16 ± 30.90	<0.01**	4.54

* *P* ≤ 0.05.

** *P <* 0.01.

LVEDV, left ventricle end diastolic volume; LVESV, left ventricle end systolic volume; LVEF, left ventricular ejection fraction; CI, cardiac index; CO, cardiac output; LVSV, left ventricular stroke volume; ECV, extracellular volume.

### 3.3 Comparison of the cardiac function, native T1 values, and ECV among different subgroups

**Figure 2:**
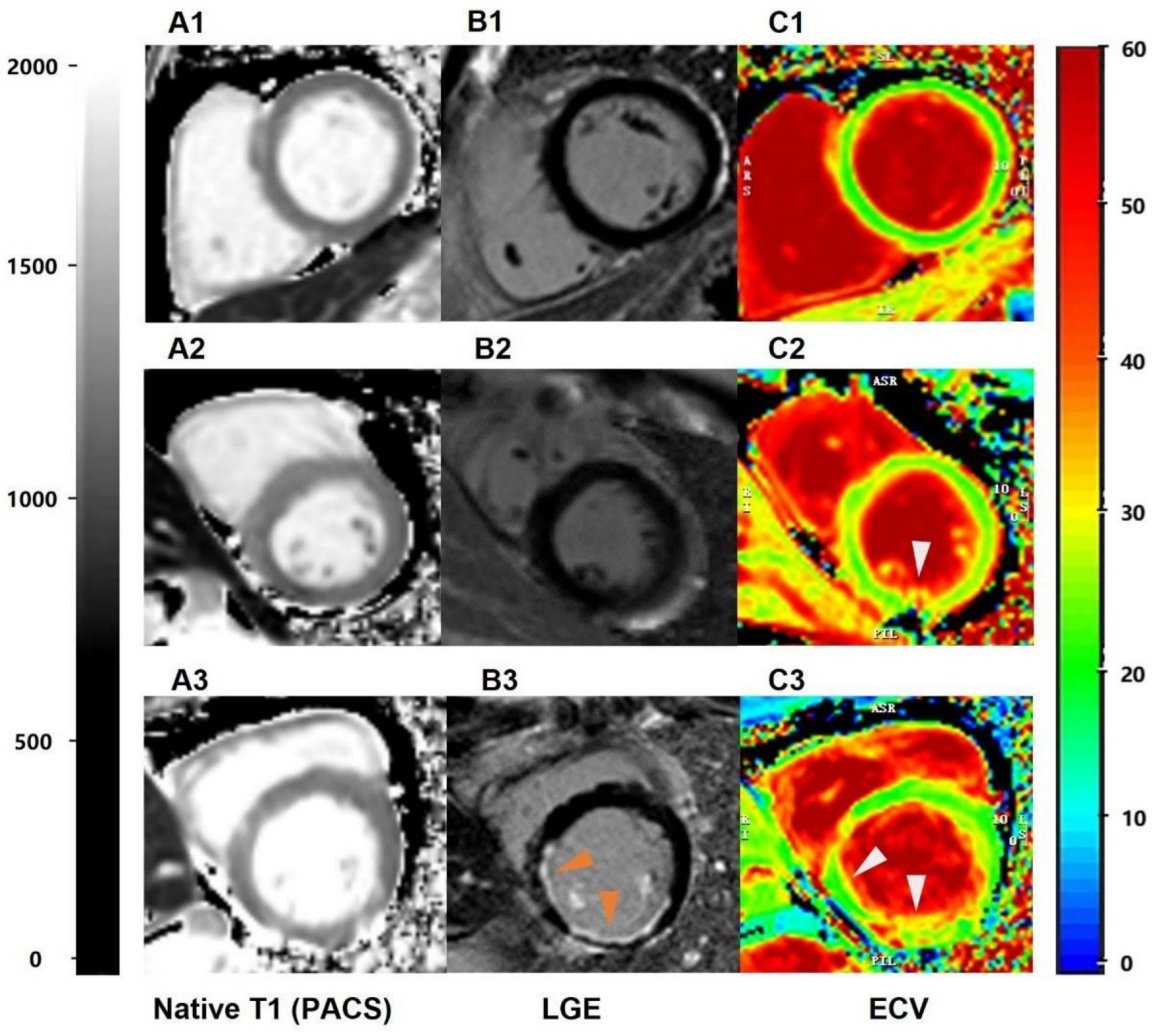
The performance of scanner-generated native T1 values in identifying different groups of patients with left ventricular myocardial short-axis CMR. (A1–A3) = scanner-generated native T1 values measured via PACS. A1 represents the native T1 value of normal subjects, with an average value of 1,250 ms. A2 represents the native T1 value of LGE− patients, with an average value of 1,320 ms. A3 represents the native T1 values of LGE+ patients, with an average value of 1,380 ms; (B1–B3) = late gadolinium enhancement (PACS), (C1–C3) = ECV obtained via the CVI post-processing software.

There was no statistically significant difference in CO and CI between the LGE+ group and the LGE− group subgroups (*P* > 0.05). In the LGE+ group, the values of LVEDV, LVESV, LVSV, ECV, and native T1 were significantly higher than those in the LGE− group (*P* < 0.05). The value of LVEF in LGE+ group was lower than that in LGE− group (*P* < 0.05). The ECV value of the LGE+ group and the LGE− group were 31.34 ± 1.93 and 28.17 ± 2.43, respectively, and the scanner-generated native T1 value were 1,300.22 ± 25.86 and 1,271.83 ± 37.79, respectively (Table 4). Typical examples were illustrated in Figure 2.

**Table 4:** Comparison of cardiac function, native T1 value and ECV between LGE+ group and LGE− group.

Parameters	LGE+ (*n* = 30)	LGE− (*n* = 30)	*P*	*t*
LVEDV (ml, $\bar{x}\pm s$)	129.62 ± 35.6	100.1 ± 22.44	<0.01**	3.84
LVESV (ml, $\bar{x}\pm s$)	62.51 ± 29.6	43.02 ± 17.73	<0.01**	3.10
LVSV (ml, $\bar{x}\pm s$)	66.76 ± 16.92	57.1 ± 13.30	0.02*	2.50
CO (L/min, $\bar{x}\pm s$)	4.63 ± 1.24	4.16 ± 1.17	0.14	1.50
CI (L/min/m², $\bar{x}\pm s$)	2.68 ± 0.88	2.36 ± 0.74	0.13	1.52
LVEF (%, $\bar{x}\pm s$)	51.37 ± 9.70	57.23 ± 9.85	0.02*	−2.32
ECV (%, $\bar{x}\pm s$)	31.34 ± 1.93	28.17 ± 2.43	<0.01**	5.60
Native T1 (ms, $\bar{x}\pm s$)	1,300.22 ± 25.86	1,271.83 ± 37.79	<0.01**	3.40

* *P <* 0.05.

** *P <* 0.01.

LVEDV, left ventricle end diastolic volume; LVESV, left ventricle end systolic volume; LVEF, left ventricular ejection fraction; CI, cardiac index; CO, cardiac output; LVSV, left ventricular stroke volume; ECV, extracellular volume.

### 3.4 Analysis of diagnostic efficacy, optimal threshold, and correlation with ECV values of native T1 among subgroups

**Figure 3:**
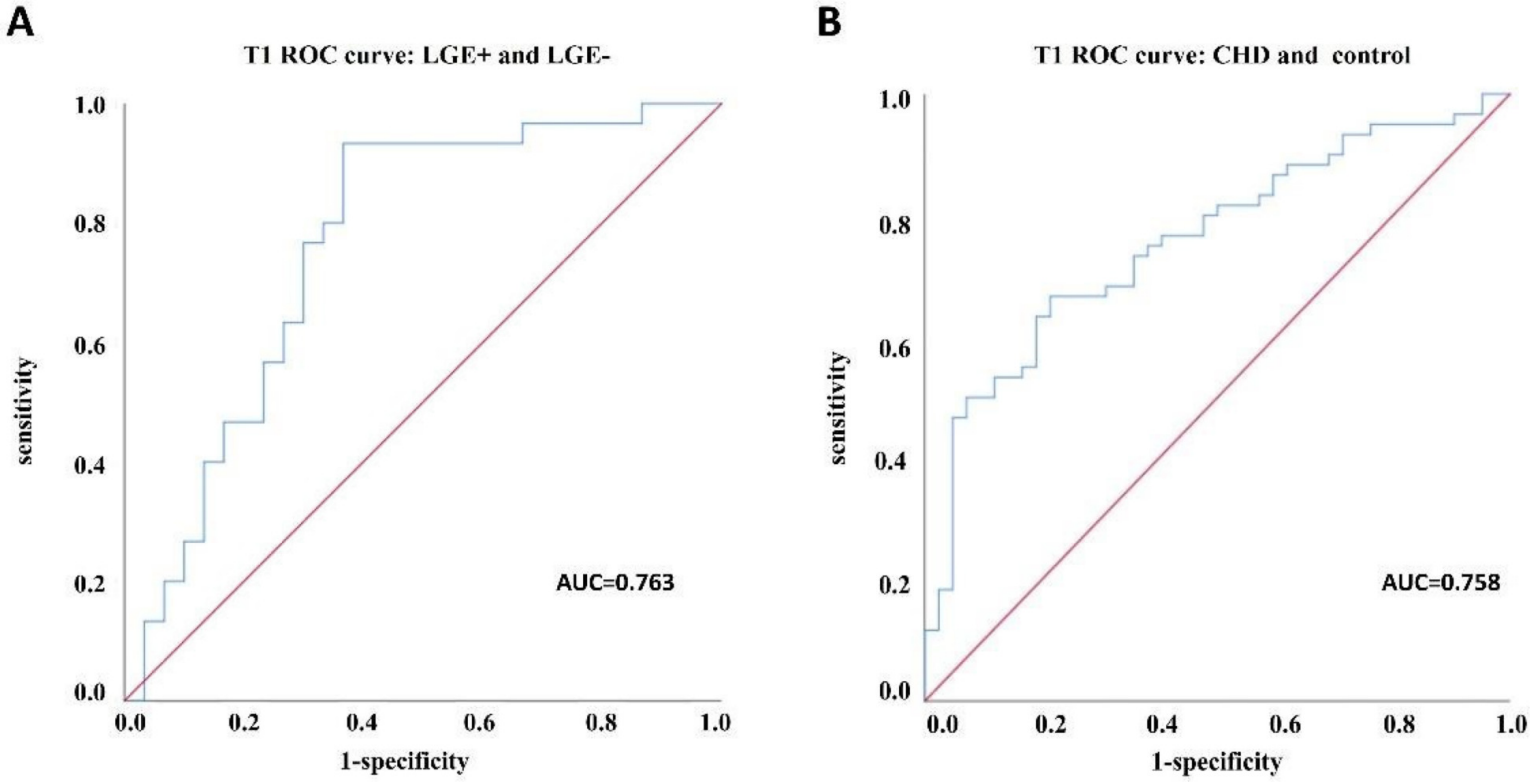
ROC curve analysis between subgroups. **(A)** ROC curve analysis between LGE+ and LGE− groups; **(B)** ROC curve analysis of CHD group and healthy control group.

**Figure 4:**
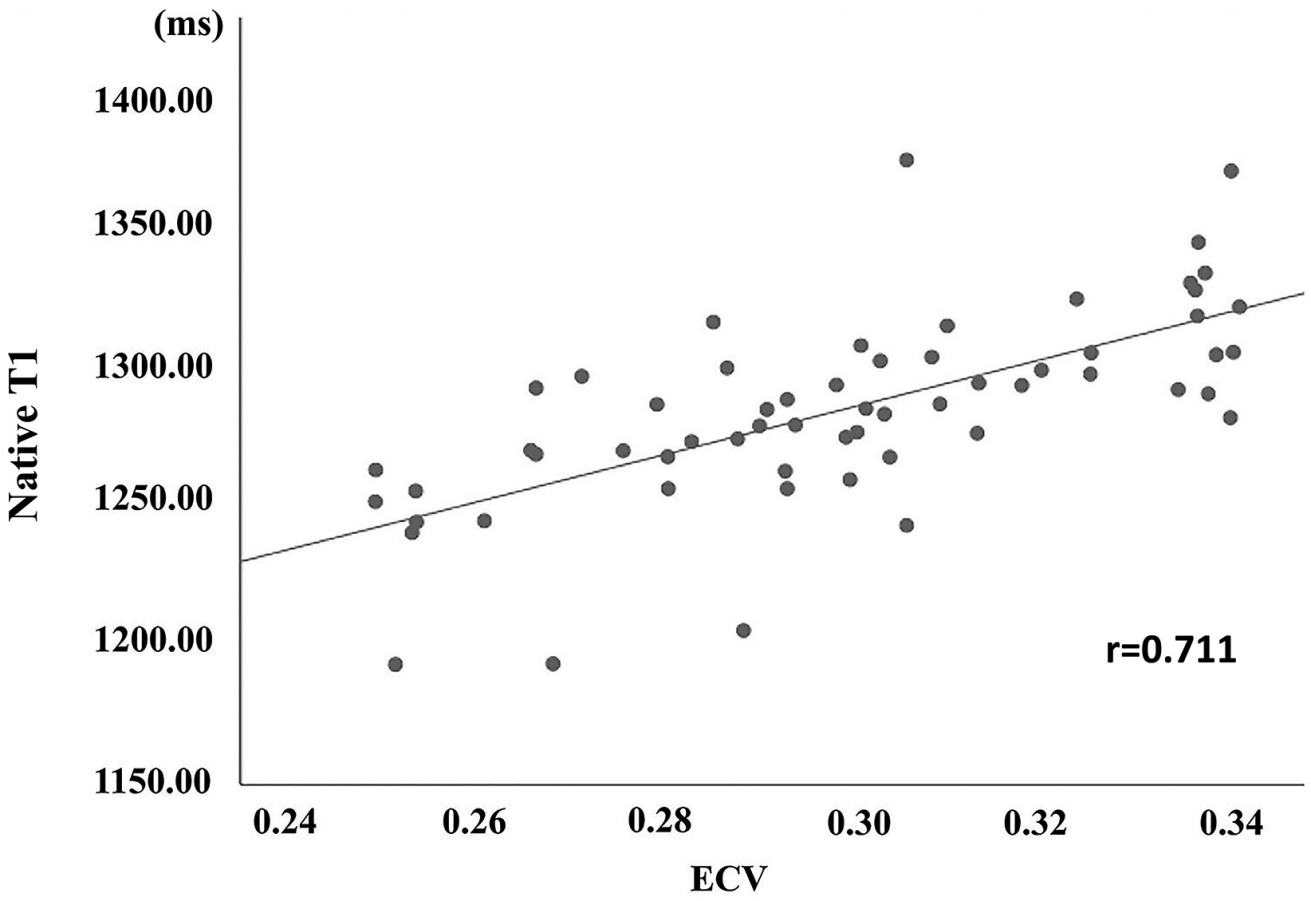
The correlation of scanner-generated native T1 and ECV between the CHD group.

For subgroups of LGE+ and LGE−, the area under ROC curve of scanner-generated native T1 was 0.763, the optimal threshold of native T1 was 1,275.50 ms, the sensitivity was 93.3%, and the specificity was 63.3% (Figure 3A). For CHD and normal groups, the area under ROC curve of native T1 was 0.758. The optimal threshold of native T1 was 1,275.07 ms, the sensitivity was 66.7%, and the specificity was 78.6% (Figure 3B). Finally, the value of scanner-generated native T1 is positively correlated with the value of ECV, *r* = 0.711 (Figure 4).

## 4 Discussion

We assessed the feasibility of this approach to evaluate myocardial diffuse fibrosis by examining the correlation between scanner-generated native T1 values measured directly via PACS workstations and ECV values obtained using heart-specific software. In this study, we demonstrated that patients with CHD without significant myocardial infarction already exhibited varying degrees of diffuse fibrosis. Moreover, a higher degree of diffuse fibrosis was observed in patients with focal myocardial infarction. Additionally, we established a diagnostic threshold range for scanner-generated native T1 values in CHD-related focal myocardial infarction. This method is non-invasive, non-contrast-agent-based, and easy to implement, representing an effective approach for evaluating myocardial diffuse fibrosis.

The scanner-generated native T1 value measured by PACS are well-suited for assessing the varying degrees of diffuse myocardial fibrosis in patients with CHD. In our study, we found a significant correlation between the scanner-generated nativeT1mapping values measured by PACS and ECV from CVI42 (*r* = 0.711). This finding aligns with previous studies examining non-CHD populations. Puntmann et al. (25) believed that nativeT1 was more effective than ECV in detecting the degree of fibrosis in patients with cardiomyopathy. However, Cui et al. (26) pointed out that ECV can distinguish between HCM and dilated cardiomyopathy (DCM), and its efficacy was superior to native T1. The discrepancies observed among various studies may be attributed to the diverse scanning equipment employed by researchers, as well as the varying sensitivity of different sequences and scanning protocols to native T1 and ECV measurements (27, 28). The corrected deep learn-based nativeT1 and ECV values are least affected by the order, supplier, and type of contrast agent used (29, 30). Additionally, consistent with findings in studies of hypertrophic cardiomyopathy (HCM) and dilated cardiomyopathy (DCM), myocardial native T1 values varied across different disease end stages. In our study, we also observed that the scanner-generated native T1 value in the LGE+ group with focal myocardial infarction was significantly higher than that in the LGE− group (1,300.22 ± 25.86 ms vs. 1,271.83 ± 37.79 ms, *p* < 0.05). Previous studies on dilated cardiomyopathy (DCM) have demonstrated that cardiomyopathy segments with late gadolinium enhancement (LGE+) exhibit significantly higher native T1 and ECV values compared to LGE− segments or healthy controls. Additionally, in comparison with healthy controls, DCM patients with LGE− segments showed significantly elevated myocardial native T1 and ECV values (31). It has been reported in the literature that when fibrosis reaches a certain level, dense collagen deposition, i.e., collagen scar tissue, will be formed, showing positive signs of LGE on magnetic resonance imaging. This may be related to the differences in collagen accumulation and changes in collagen components in different stages of myocardial interstitial fibrosis (32).

At the same time, we also concluded that the threshold of scanner-generated nativeT1 value in the CHD group with myocardial infarction was 1,275.50. And the threshold changes were not significant among subgroups, but the sensitivity differences were quite apparent. The sensitivity of native T1 values was significantly higher between the subgroups of LGE+ and LGE− compared to the normal and CHD groups. Previous studies (33) showed that patients with hypertrophic cardiomyopathy (HCM) with adverse endpoints were 1,316.6 ± 112.5 ms, while those with HCM without adverse endpoints were 1,278.4 ± 64.6 ms (34). Similarly, DCM patients exhibited longer mean native T1 values (1,283.44 ± 72.10 ms vs. 1,221.80 ± 58.80 ms) and a higher mean myocardial ECV values (42.59 ± 7.40% vs. 32.24 ± 5.01%) compared to healthy participants (31). These are all attempts to use plain scans to initially predict the occurrence of adverse events.

Several major limitations must be considered when interpreting this study. Firstly, the lack of myocardial histopathological sections as a standard for confirming the presence of diffuse fibrosis. That may have had an impact on the results. Secondly, the relatively small sample size may have caused an underestimation of fibrosis process and prognosis in the coronary heart disease patients. Thirdly, initially in our experiment, we did not compare the consistency between the native T1 values derived from the post-processing software and those generated by the scanner. Going forward, we will increase the sample size to conduct a consistency study between the two.

To sum up, directly measuring scanner-generated native T1 values through PACS, which eliminates the need for additional contrast agent injection and the purchase of specialized cardiac post-processing software, emerges as an eminently viable and cost-effective alternative. This approach is particularly suitable for patients with impaired renal function or allergies to contrast agents, as well as for institutions lacking third-party post-processing software. It holds significant clinical implications for the assessment of diffuse myocardial fibrosis.

## Data Availability

The original contributions presented in the study are included in the article/Supplementary Material, further inquiries can be directed to the corresponding authors.

## References

[1] Bevan G Pandey A Griggs S Dalton JE Zidar D Patel S Neighborhood-level social vulnerability and prevalence of cardiovascular risk factors and coronary heart disease. Curr Probl Cardiol. (2023) 48(8):101182. 10.1016/j.cpcardiol.2022.101182 35354074 PMC9875801

[2] Reynolds HR Shaw LJ Min JK Spertus JA Chaitman BR Berman DS Association of sex with severity of coronary artery disease, ischemia, and symptom burden in patients with moderate or severe ischemia: secondary analysis of the ISCHEMIA randomized clinical trial. JAMA Cardiol. (2020) 5(7):773–86. 10.1001/jamacardio.2020.0822 32227128 PMC7105951

[3] Liu D Borlotti A Viliani D Jerosch-Herold M Alkhalil M De Maria GL CMR native T1 mapping allows differentiation of reversible versus irreversible myocardial damage in ST-segment-elevation myocardial infarction: an OxAMI study (Oxford Acute Myocardial Infarction). Circ Cardiovasc Imaging. (2017) 10(8):e005986. 10.1161/CIRCIMAGING.116.005986 28798137 PMC5555391

[4] DiLorenzo MP Grosse-Wortmann L. Myocardial fibrosis in congenital heart disease and the role of MRI. Radiol Cardiothorac Imaging. (2023) 5(3):e220255. 10.1148/ryct.220255 37404787 PMC10316299

[5] Schmeckpeper J Kim K George SA Blackwell DJ Brennan JA Efimov IR Ryr2 inhibition with dantrolene is antiarrhythmic, prevents further pathological remodeling, and improves cardiac function in chronic ischemic heart disease. J Mol Cell Cardiol. (2023) 181:67–78. 10.1016/j.yjmcc.2023.05.009 37285929 PMC10526741

[6] Karamitsos TD Arvanitaki A Karvounis H Neubauer S Ferreira VM. Myocardial tissue characterization and fibrosis by imaging. JACC Cardiovasc Imaging. (2020) 13(5):1221–34. 10.1016/j.jcmg.2019.06.030 31542534

[7] Lai W Chen-Xu Z Jian-Xun D Jie H Ling-Cong K Dong-Ao-Lei A Prognostic implications of left ventricular torsion measured by feature-tracking cardiac magnetic resonance in patients with ST-elevation myocardial infarction. Eur Heart J Cardiovasc Imaging. (2023) 24(6):785–95. 10.1093/ehjci/jeac177 36056877

[8] Del Buono MG Garmendia CM Seropian IM Gonzalez G Berrocal DH Biondi-Zoccai G Heart failure after ST-elevation myocardial infarction: beyond left ventricular adverse remodeling. Curr Probl Cardiol. (2023) 48(8):101215. 10.1016/j.cpcardiol.2022.101215 35460680

[9] Thavendiranathan P Shalmon T Fan CS Houbois C Amir E Thevakumaran Y Comprehensive cardiovascular magnetic resonance tissue characterization and cardiotoxicity in women with breast cancer. JAMA Cardiol. (2023) 8(6):524–34. 10.1001/jamacardio.2023.0494 37043251 PMC10099158

[10] Si D Wu Y Xiao J Qin X Guo R Liu B Three-dimensional high-resolution dark-blood late gadolinium enhancement imaging for improved atrial scar evaluation. Radiology. (2023) 307(5):e222032. 10.1148/radiol.222032 37278633

[11] She J Zhao S Chen Y Zeng M Jin H. Detecting regional fibrosis in hypertrophic cardiomyopathy: the utility of myocardial strain based on cardiac magnetic resonance. Acad Radiol. (2023) 30(2):230–8. 10.1016/j.acra.2022.03.022 35469720

[12] Alachkar MN Mischke T Mahnkopf C. Cardiac magnetic resonance imaging and the myocardium: differentiation between vital and nonvital tissue. Herzschrittmacherther Elektrophysiol. (2022) 33(3):272–7. 10.1007/s00399-022-00874-8 35781833

[13] Kellman P Hansen MS. T1-mapping in the heart: accuracy and precision. J Cardiovasc Magn Reson. (2014) 16(1):2. 10.1186/1532-429X-16-2 24387626 PMC3927683

[14] Axelsson Raja A Farhad H Valente AM Couce JP Jefferies JL Bundgaard H Prevalence and progression of late gadolinium enhancement in children and adolescents with hypertrophic cardiomyopathy. Circulation. (2018) 138(8):782–92. 10.1161/CIRCULATIONAHA.117.032966 29622585 PMC6173664

[15] Foussier C Barral PA Jerosh-Herold M Gariboldi V Rapacchi S Gallon A Quantification of diffuse myocardial fibrosis using CMR extracellular volume fraction and serum biomarkers of collagen turnover with histologic quantification as standard of reference. Diagn Interv Imaging. (2021) 102(3):163–9. 10.1016/j.diii.2020.07.005 32830084

[16] Puntmann VO Carr-White G Jabbour A Yu CY Gebker R Kelle S Native T1 and ECV of noninfarcted myocardium and outcome in patients with coronary artery disease. J Am Coll Cardiol. (2018) 71(7):766–78. 10.1016/j.jacc.2017.12.020 29447739

[17] Yang EY Ghosn MG Khan MA Gramze NL Brunner G Nabi F Myocardial extracellular volume fraction adds prognostic information beyond myocardial replacement fibrosis. Circ Cardiovasc Imaging. (2019) 12(12):e009535. 10.1161/CIRCIMAGING.119.009535 31838882 PMC7529265

[18] deFilippi CR Tran H Gattani R Daniels LB Shah P Ilkhanoff L Association of cardiac troponin T and growth differentiation factor 15 with replacement and interstitial cardiac fibrosis in community dwelling adults: the multi-ethnic study of atherosclerosis. Front Cardiovasc Med. (2023) 10:1104715. 10.3389/fcvm.2023.1104715 36844723 PMC9949377

[19] Lewis GA Rosala-Hallas A Dodd S Schelbert EB Williams SG Cunnington C Impact of myocardial fibrosis on cardiovascular structure, function and functional Status in heart failure with preserved ejection fraction. J Cardiovasc Transl Res. (2022) 15(6):1436–43. 10.1007/s12265-022-10264-7 35790651 PMC9722869

[20] Abadia AF Aquino GJ Schoepf UJ Wels M Schmidt B Sahbaee P Automated dual-energy computed tomography-based extracellular volume estimation for myocardial characterization in patients with ischemic and nonischemic cardiomyopathy. J Thorac Imaging. (2022) 37(5):307–14. 10.1097/RTI.0000000000000656 35475983

[21] Shang Y Zhang X Zhou X Wang J. Extracellular volume fraction measurements derived from the longitudinal relaxation of blood-based synthetic hematocrit may lead to clinical errors in 3T cardiovascular magnetic resonance. J Cardiovasc Magn Reson. (2018) 20(1):56. 10.1186/s12968-018-0475-6 30089499 PMC6083590

[22] Chen W Doeblin P Al-Tabatabaee S Klingel K Tanacli R Jakob Weiß K Synthetic extracellular volume in cardiac magnetic resonance without blood sampling: a reliable tool to replace conventional extracellular volume. Circ Cardiovasc Imaging. (2022) 15(4):e013745. 10.1161/CIRCIMAGING.121.013745 35360924 PMC9015035

[23] Patel MR Calhoon JH Dehmer GJ Grantham JA Maddox TM Maron DJ ACC/AATS/AHA/ASE/ASNC/SCAI/SCCT/STS 2016 appropriate use criteria for coronary revascularization in patients with acute coronary syndromes: a report of the American College of Cardiology appropriate use criteria task force, American association for thoracic surgery, American heart association, American society of echocardiography, American society of nuclear cardiology, society for cardiovascular angiography and interventions, society of cardiovascular computed tomography, and the society of thoracic surgeons. J Nucl Cardiol. (2017) 24(2):439–63. 10.1007/s12350-017-0780-8 28265967

[24] Neisius U El-Rewaidy H Nakamori S Rodriguez J Manning WJ Nezafat R. Radiomic analysis of myocardial native T (1) imaging discriminates between hypertensive heart disease and hypertrophic cardiomyopathy. JACC Cardiovasc Imaging. (2019) 12(10):1946–54. 10.1016/j.jcmg.2018.11.024 30660549 PMC7032053

[25] Puntmann VO Voigt T Chen Z Mayr M Karim R Rhode K Native T1 mapping in differentiation of normal myocardium from diffuse disease in hypertrophic and dilated cardiomyopathy. JACC Cardiovasc Imaging. (2013) 6(4):475–84. 10.1016/j.jcmg.2012.08.019 23498674

[26] Cui Y Chen Y Cao Y Liu J Song J Zhang S Myocardial extracellular volume fraction measurements with MOLLI 5(3)3 by cardiovascular MRI for the discrimination of healthy volunteers from dilated and hypertrophic cardiomyopathy patients. Clin Radiol. (2019) 74(9):732.e9–e16. 10.1016/j.crad.2019.04.019 31122714

[27] Treibel TA Fontana M Maestrini V Castelletti S Rosmini S Simpson J Automatic measurement of the myocardial interstitium: synthetic extracellular volume quantification without hematocrit sampling. JACC Cardiovasc Imaging. (2016) 9(1):54–63. 10.1016/j.jcmg.2015.11.008 26762875

[28] Vo HQ Marwick TH Negishi K. Pooled summary of native T1 value and extracellular volume with MOLLI variant sequences in normal subjects and patients with cardiovascular disease. Int J Cardiovasc Imaging. (2020) 36(2):325–36. 10.1007/s10554-019-01717-3 31686277

[29] Suh YJ Kim PK Park J Park EA Jung JI Choi BW. Phantom-based correction for standardization of myocardial native T1 and extracellular volume fraction in healthy subjects at 3-tesla cardiac magnetic resonance imaging. Eur Radiol. (2022) 32(12):8122–30. 10.1007/s00330-022-08936-8 35771246 PMC9705515

[30] Zhi Y Gui FD Xue M Long YT Miao W Yi Y Focal ischemic myocardial fibrosis assessed by late gadolinium enhancement cardiovascular magnetic resonance in patients with hypertrophic cardiomyopathy. BMC Cardiovasc Disord. (2024) 24(1):203. 10.1186/s12872-024-03859-2 38594610 PMC11003119

[31] Gao Y Wang HP Liu MX Gu H Yuan XS Biekan J Early detection of myocardial fibrosis in cardiomyopathy in the absence of late enhancement: role of T1 mapping and extracellular volume analysis. Eur Radiol. (2023) 33(3):1982–91. 10.1007/s00330-022-09147-x 36241919

[32] Su MY Lin LY Tseng YH Chang CC Wu CK Lin JL CMR-verified diffuse myocardial fibrosis is associated with diastolic dysfunction in HFpEF. JACC Cardiovasc Imaging. (2014) 7(10):991–7. 10.1016/j.jcmg.2014.04.022 25240451

[33] Li Y Liu X Yang F Wang J Xu Y Fang T Prognostic value of myocardial extracellular volume fraction evaluation based on cardiac magnetic resonance T1 mapping with T1 long and short in hypertrophic cardiomyopathy. Eur Radiol. (2021) 31(7):4557–67. 10.1007/s00330-020-07650-7 33449190

[34] Zhang Y Zhang X Wang Y Hu X Wang B Yang J Relationship between diffuse fibrosis assessed by CMR and depressed myocardial strain in different stages of heart failure. Eur J Radiol. (2023) 164:110848. 10.1016/j.ejrad.2023.110848 37156180

